# Conformational Rearrangements in the Redox Cycling of NADPH-Cytochrome P450 Reductase from *Sorghum bicolor* Explored with FRET and Pressure-Perturbation Spectroscopy

**DOI:** 10.3390/biology11040510

**Published:** 2022-03-25

**Authors:** Bixia Zhang, ChulHee Kang, Dmitri R. Davydov

**Affiliations:** Department of Chemistry, Washington State University, Pullman, WA 99164, USA; bixia.zhang@wsu.edu

**Keywords:** cytochrome P450 reductase, conformational change, FRET, pressure-perturbation spectroscopy, protein hydration, reduction kinetics, stop-flow spectroscopy, *Sorghum bicolor*

## Abstract

**Simple Summary:**

NADPH-cytochrome P450 reductase (CPR) enzymes are known to undergo an ample conformational transition between the closed and open states in the process of their redox cycling. To explore the conformational landscape of CPR from the potential biofuel crop *Sorghum bicolor* (SbCPR), we incorporated a FRET donor/acceptor pair into the enzyme and employed rapid scanning stop-flow and pressure perturbation spectroscopy to characterize the equilibrium between its open and closed states at different stages of the redox cycle. Our results suggest the presence of several open conformational sub-states differing in the system volume change associated with the opening transition (Δ*V*^0^). Although the closed conformation always predominates in the conformational landscape, the population of the open conformations increases by order of magnitude upon the two-electron reduction and the formation of the disemiquinone state of the enzyme. In addition to elucidating the functional choreography of plant CPRs, our study demonstrates the high exploratory potential of a combination of the pressure-perturbation approach with the FRET-based monitoring of protein conformational transitions.

**Abstract:**

NADPH-cytochrome P450 reductase (CPR) from *Sorghum bicolor* (SbCPR) serves as an electron donor for cytochrome P450 essential for monolignol and lignin production in this biofuel crop. The CPR enzymes undergo an ample conformational transition between the closed and open states in their functioning. This transition is triggered by electron transfer between the FAD and FMN and provides access of the partner protein to the electron-donating FMN domain. To characterize the electron transfer mechanisms in the monolignol biosynthetic pathway better, we explore the conformational transitions in SbCPR with rapid scanning stop-flow and pressure-perturbation spectroscopy. We used FRET between a pair of donor and acceptor probes incorporated into the FAD and FMN domains of SbCPR, respectively, to characterize the equilibrium between the open and closed states and explore its modulation in connection with the redox state of the enzyme. We demonstrate that, although the closed conformation always predominates in the conformational landscape, the population of open state increases by order of magnitude upon the formation of the disemiquinone state. Our results are consistent with several open conformation sub-states differing in the volume change (Δ*V*^0^) of the opening transition. While the Δ*V*^0^ characteristic of the oxidized enzyme is as large as −88 mL/mol, the interaction of the enzyme with the nucleotide cofactor and the formation of the double-semiquinone state of CPR decrease this value to −34 and −18 mL/mol, respectively. This observation suggests that the interdomain electron transfer in CPR increases protein hydration, while promoting more open conformation. In addition to elucidating the functional choreography of plant CPRs, our study demonstrates the high exploratory potential of a combination of the pressure-perturbation approach with the FRET-based monitoring of protein conformational transitions.

## 1. Introduction

Cytochromes P450, the heme-thiolate enzymes found in all domains of life, from *Eubacteria* and *Archaea* to *Eukarya*, probably appeared about 3.5 billion years ago [1], when the oxygen content in the atmosphere was negligible. It is suggested that ancient cytochromes P450 acted as reducing enzymes and could play the role of NO reductases [2]. When green plants began to release oxygen into the atmosphere about 2 billion years ago, cytochromes P450 became involved in the synthesis and oxidative metabolism of fatty acids and steroids [3]. Later, the catalytic oxidation of hydrophobic compounds became the primary function of cytochromes P450.

Nowadays, cytochromes P450 act as terminal oxidases in monooxygenase systems, oxidizing various exogenous and endogenous substrates. They are involved in the oxidative metabolism and detoxification of low molecular weight foreign lipophilic compounds (xenobiotics) as well as in the synthesis of pigments, hormones, second messengers, antibiotics, and toxins. To perform these functions better, the eukaryotic P450s became membrane incorporated. In most cases, they are associated with the membranes of the endoplasmic reticulum (ER), where they interact with their partner proteins via lateral diffusion and the formation of dissociative complexes.

All known eukaryotic cytochromes P450 and most bacterial analogs are not self-sufficient in their catalytic function. The monooxygenase reaction requires two electrons, which are usually transferred to cytochrome P450 from a protein partner. For most eukaryotic cytochromes P450, the role of electron donor is played by NADPH-cytochrome P450 reductase (CPR), a flavoprotein that contains two flavin cofactors, FAD and FMN. These flavins are situated in distinct protein domains termed FAD and FMN domains. These two domains are connected with a flexible connecting loop. The reducing equivalents from NADPH are first acquired by FAD and then transferred to FMN, which serves as an ultimate electron donor for P450.

While the majority of animal cytochrome P450 species are involved in xenobiotic metabolism, the predominant part of the plant P450s participates in biosynthetic pathways. They play a critical role in synthesizing lignin, UV protectants, pigments, defense compounds, fatty acids, hormones, and secondary messengers. In particular, cytochrome-P450-dependent cinnamate-4-hydroxylase (C4H), *p*-coumaroyl quinate/shikimate-3’-hydroxylase (C3′H), and ferulate-5-hydroxylase (F5H) are the critical branching points in the phenylpropanoid metabolizing pathway, which is required for the biosynthesis of monolignol and serves as a starting point for the production of flavonoids, coumarins, lignans, and lignin.

This investigation represents a part of our studies aimed at elucidating the mechanisms of function and regulation of phenylpropanoid-metabolizing monooxygenases from *Sorghum bicolor*, a U.S. strategic plant for biofuel production [4,5,6]. The detailed mechanistic knowledge of these enzymes will enable specific manipulation of lignin composition and content and thus economize the industrial cost of biofuel production. Furthermore, the high flexibility of the hinge region of SbCPR demonstrated in our previous report [7] offers potential for manipulating the functional properties of the enzyme through rational engineering in this region. The present study explores the functional mechanisms of NADPH-cytochrome P450 reductase 2b, one of the three CPR enzymes in *Sorghum bicolor* serving as electron donors for C4H, C3′H, and F5H P450 enzymes. This enzyme is referred to as SbCPR from now on.

**Scheme 1:**
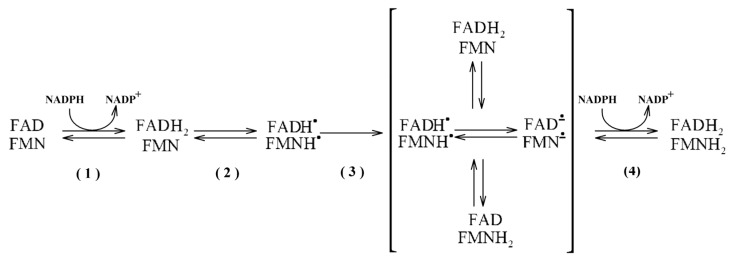
Scheme of electron transfer events in CPR.

In general, the functional redox cycling in SbCPR enzymes follows the scheme common to all known CPRs. Their reduction from the completely oxidized (FAD, FMN) to the four-electron reduced state (FADH_2_, FMNH_2_) includes (1) hydride transfer from NADPH to FAD, resulting in a two-electron reduced (FADH_2_, FMN) state; (2) inter-flavin electron transfer from FADH_2_ to FMN with the formation of the neutral (blue) disemiquinone (FADH^•^, FMNH^•^); (3) establishing a transient equilibrium between the latter and the other two-electron reduced states (FAD, FMNH_2_), (FADH_2_, FMN) and anionic semiquinones (FAD^$\underline{•}$^, FMN^$\underline{•}$^); (4) supply of another pair of electrons from NADPH yielding the four-electron reduced (FADH_2_, FMNH_2_) state. This sequence of events is illustrated in Scheme 1.

In most eukaryotic monooxygenases, this four-electron reduced state is believed to serve as the P450 electron donor so that FMN cofactor interchanges between the hydroquinone and semiquinone states. By contrast, the FMN moiety in the bacterial CPR-P450 chimera P450BM-3 shuttles between the semiquinone and oxidized states instead [8]. However, in all cases, the transfer of electrons between the FAD and FMN domains remains an obligatory step in the CPR redox cycle.

In this perspective, understanding electron transfer mechanisms and the respective conformational rearrangements has become a challenge for researchers. In the first solved CPR structure [9] and several subsequently published structures of CPR from various species [10,11,12], the distance between FAD and FMN is around 4 Å, which is considered favorable for the inter-flavin electron transfer. However, this proximity of the two domains does not allow the electron acceptor protein to reach FMN. This circumstance and the largely disordered structure of the connecting loop brought forward a hypothesis of a large-scale opening-and-closing transition involved in the CPR electron transfer mechanism.

In further X-ray crystallographic studies, the CPR variant with a shortened connecting loop was found locked in the open state, which is flawed in terms of inter-flavin electron transfer, but effective in the transfer from FMN to the heme [13]. In contrast, a variant of rat CPR where the two domains are interconnected with a disulfide bond [14] was found locked in the closed conformation. These and other resolved CPR structures [11,12,15,16] demonstrated exceptional conformational flexibility of CPR and emphasized the pivotal functional role of the transitions between the enzyme’s closed and open states.

The conformational landscape of CPR and its relevance to the redox cycling of the enzyme was further explored with a wide variety of biophysical techniques ranging from NMR [17] and small-angle neutron and X-ray scattering (SANS and SAX, [18,19,20,21]) to ion mobility mass spectrometry [22] and single-molecule fluorescence resonance energy transfer (FRET [23,24,25,26]). Despite some contradictory observations in these studies, all of them are consistent in demonstrating a transition of the closed state of the enzyme into an open conformation upon the interdomain electron transfer event and the formation of the disemiquinone state (see [27] for a review). At the same time, these studies also demonstrated that the initial two-state model is insufficient to adequately depict the conformational landscape of CPR.

According to the current concepts, instead of being represented by two discrete states, the enzyme exists in a dynamic equilibrium among multiple conformations differing in the relative positioning of the FMN and FAD domains [27]. Most available data suggest that the closed conformations predominate in the completely oxidized CPR [18,19,20,21,24,26]. Some studies also suggest the further displacement of the conformational equilibrium towards the closed state upon the binding of NADPH to the oxidized enzyme [25,27]. There are also strong indications of the predominance of the closed conformations in the four-electron reduced enzyme [20,21,23,26].

Despite a foreseeable similarity of CPR enzymes from different organisms in general mechanisms of electron transport and the related protein choreography, the enzymes from different kingdoms of life may differ considerably in the kinetic and thermodynamic parameters of the individual steps of the redox cycle. Thus, the structure study of *Arabidopsis thaliana* suggests that the oxidized state of plant CPR enzymes may have considerably more open conformation than that characteristic of their mammalian counterparts [11]. Furthermore, the studies of the kinetics of electron transfer in plant CPR demonstrate that the rate of electron transfer from NADPH to FAD in these enzymes is over 50 times faster than in their mammalian counterparts [28].

In the present study, we explore the conformational equilibrium in SbCPR and its modulation during the redox cycle of the enzyme with the use of a combination of the FRET-based detection of protein conformational rearrangements with the rapid scanning absorbance and fluorescence stop-flow technique and the pressure-perturbation approach. While the FRET-based methods and rapid scanning stop-flow techniques have already been applied in both mammalian [24,25,27] and plant [26] CPR studies, the present study represents the first attempt to explore the conformational landscape of CPR with pressure perturbation.

In pressure-perturbation studies, hydrostatic pressure is a variable parameter affecting the protein conformational landscapes. Along with the effects of temperature, varying pressure is indispensable for a detailed understanding of the mechanisms of protein conformational transitions. The basis of pressure effects is the change in system volume that accompanies biochemical processes [29,30,31,32]. According to Le Chatelier’s principle, increased pressure enhances processes accompanied by a decrease in system volume and, conversely, inhibits processes occurring with a volume increase. A prevalent part of the volume changes in protein transitions stems from the changes in interactions with solvents [33,34,35,36,37,38]. These include water penetration into the cavities and water constriction around solvent-exposed polar groups of the protein [31,38,39,40,41,42,43]. Thus, the volume change resulting from the penetration of one water molecule into a protein cavity is equal to −18 mL/mol, while the solvation of a singly charged ion in water is characterized by Δ*V* values of the order of −10 mL/mol [31]. Generally speaking, pressure increase enhances protein hydration, which therefore constitutes the core of pressure-induced protein transitions [33,34,37,43,44,45,46].

Ample conformational transitions necessary for CPR redox cycling are implied to be associated with significant changes in the protein–solvent interactions. The process of protein opening is reported to involve the breaking of several salt bridges [11,12,24] and the subsequent hydration of the newly exposed charges on the protein surface. Therefore, pressure perturbation is the method of choice for exploring the CPR conformational landscape. It allows to judge the changes in protein hydration in the redox cycling of the enzyme and provides a simple means for determining the position of equilibria in the system of open and closed conformation at its different redox states.

To make the studies possible, we incorporate a FRET donor/acceptor pair into the FAD and FMN domains of SbCPR. Our study demonstrates that, although the closed conformation always predominates in the conformational landscape, the population of open state increases by order of magnitude upon the formation of the disemiquinone state. Our results are consistent with several open conformation sub-states differing in the opening transition volume change (Δ*V*^0^). The details of the SbCPR electron transfer mechanism revealed in this study will provide vital information for engineering the monolignol pathway and subsequent lignin polymerization in order to improve the use of *Sorghum bicolor* as a biofuel plant. In addition to elucidating the functional choreography of plant CPRs, our study demonstrates the high exploratory potential of a combination of the pressure-perturbation approach with the FRET-based monitoring of protein conformational dynamics.

## 2. Materials and Methods

### 2.1. Materials

DY-520XL and DY-731 were the products of Dyomics GMBH (Jena, Germany). Monobromobimane (MBBr) was obtained from Invitrogen/Molecular Probes (Eugene, OR, USA), now a part of ThermoFisher Scientific. Igepal CO-630, glucose oxidase, catalase, NADPH, glucose-6-phosphate, and glucose-6-phosphate-dehydrogenase were obtained from Sigma-Aldrich (St. Louis, MO, USA). 2′5′-ADP was purchased from Santa Cruz Biotechnology (Dallas, TX, USA). All other chemicals were of the highest grade commercially available and were used without further purification.

### 2.2. Cloning, Protein Expression, and Purification

The SbCPR cDNA corresponding to the *Sorghum bicolor* gene SORBI_3007G088000 was modified with a truncation of its N-terminal transmembrane sequence (Δ2–50) and the addition of the C-terminal hexahistidine tag. The resulting construct was cloned into a pET-30a (+) vector. For the SbCPR C596S mutant, site-directed mutations were created in the SbCPR coding region by PCR-based amplification using Phusion High-Fidelity DNA polymerase (New England Biolabs, Ipswich, MA, USA). The amplification was performed using the forward primer CTTCGGAAGCAGAAATAGCAAGATGGACT, and the reverse primer TATTTCTGCTTCCGAAGAAGAACACGGATG was followed by *Dpn*I (New England Biolabs, Ipswich, MA, USA) digestion to remove the template strand prior to transformation to XL1-blue competent cell for amplification. C596S mutation was confirmed by DNA sequencing (Fisher Scientific, Waltham, MA, USA). The replacement was performed to limit the possible location of the incorporated fluorescent probes to Cys-235 and Cys-536 (see Section 3.1). The purification methods were the same for the wild-type and C596S mutant. The vectors were transformed into *Escherichia coli* Rosetta 2 (DE3) cells. Three liters of Lysogeny Broth medium complemented with 25 μg mL^−1^ chloramphenicol and 50 μg mL^−1^ kanamycin were inoculated with 20 mL from the culture. The cells were grown at 37 °C until the optical density of the culture at 600 nm reached 0.6~0.8. At this point, the temperature was set at 25 °C and 0.5 mM IPTG was added. After the incubation of the culture for 16 h, the cells were harvested by centrifugation at 5000 rpm for 20 min at 4 °C and resuspended in the Buffer A (50 mM Tris-HCl, 300 mM NaCl, pH 8.0) with 20 mM imidazole. After sonicating on ice for 30 min with a Model 450 sonicator (Branson Ultrasonics, Danbury, CT, USA), the cell debris was removed by ultracentrifugation. The clear lysate was loaded onto the column of Ni-NTA agarose (Qiagen, Germantown, MD, USA) and extensively washed with the same buffer. Modified CPR protein was eluted by Buffer A containing 250 mM imidazole, pH 8.0. After concentrating the protein to 2 mg mL^−1^, its solution was dialyzed against 5 mM potassium phosphate buffer and applied onto a CHT ceramic hydroxyapatite column (Bio-Rad, Hercules, CA, USA). The fraction containing SbCPR was eluted by a linear phosphate gradient and then concentrated to ~30 mg/mL. Final purity was analyzed by SDS-PAGE, and the concentration was determined by Bradford assay (Bio-Rad).

### 2.3. Incorporation of Thiol-Reactive Fluorescent Probes

In this study, we used DY-520XL and DY731 fluorescent dyes manufactured by Dyomics GMBH, Jena, Germany) as FRET donor and acceptor fluorophores. Both probes were used as maleimide derivatives (product numbers 520XL-03 and 731-03, respectively). Incorporating these probes into SbCPR involves their attachment to the thiol groups of the cysteine residues of the protein. Prior to modification, SbCPR was stored in 125 mM K-phosphate buffer, pH 7.4, containing 2 mM TCEP (Storage Buffer). TCEP was removed by passing the protein solution through a spin-out column of Bio-Gel P6 desalting resin (Bio-Rad, Hercules, CA, USA) equilibrated with 125 mM K-phosphate buffer, pH 7.4. After diluting the protein with the same buffer to the concentration of 10 µM, a 3 mM solution of DY-520XL maleimide in acetone was added to the final concentration of 10 µM, and the solution was incubated at 4 °C under continuous stirring. The increase in the fluorescence of DY-520XL at 630 nm (excitation at 520 nm) in the process of modification was monitored to ensure reaction completion. After the stabilization of fluorescence in approximately one hour of incubation, an acetone solution of the second probe (DY731 maleimide) was added to the final concentration of 10 µM. The reaction was followed by monitoring a decrease in the fluorescence of DY-520XL. The process of the second modification required 3–4 h for completion. Finally, the reaction was terminated by adding reduced glutathione to the concentration of 1 mM. The protein was concentrated to 100–200 µM and passed through a spin-out column of Bio-Gel P6 equilibrated with 125 mM potassium phosphate buffer, pH 7.4 to remove glutathione adducts unreacted dyes. The stoichiometry of labeling by DY-520XL and DY-731 was determined based on the spectrum of absorbance of the modified protein. This calculation used the extinction coefficients of 0.05 µM^−1^ cm^−1^ at 520 nm and 0.24 µM^−1^ cm^−1^ at 736 nm for DY-520XL and DY-731, respectively, as specified by the manufacturer.

### 2.4. Rapid Kinetic Studies with Absorbance and Fluorescence Spectroscopy

The kinetics of the NADPH-dependent reduction of SbCPR were studied with rapid-scanning stop-flow spectroscopy. The experiments were performed at 5 °C in 20 mM HEPES buffer, pH 7.4, containing an oxygen-scavenging system consisting of 60 mM glucose, 300 units/mL glucose oxidase, and 2000 units/mL catalase. The concentration of SbCPR and NADPH in the optical cell was equal to 20 μM and 200 μM, respectively. The solution of NADPH also contained 2 mM glucose-6-phosphate and 4 units/mL glucose-6-phosphate dehydrogenase, which were added to keep the concentration of NADPH constant. The stop-flow experiments were performed with the use of RX 2000 Rapid Mixing Stopped-flow Accessory manufactured by Applied Photophysics Ltd. (Leatherhead, Surrey, U.K.) connected to the master channel of an MC2000-2 two-channel CCD spectrometer (Ocean Optics, Inc., Dunedin, FL, USA) equipped with a custom-made thermostated cell holder and a PX-2 pulsed xenon lamp light source (Ocean Optics). The RX 2000 Accessory was custom modified to allow remote control of mixing from the data acquisition software. The absorbance spectra in the range of 320–700 nm were collected with the time intervals changing from 2 ms to 2 s per spectrum using custom data acquisition software.

Rapid kinetics of the changes in SbCPR conformation during the reduction process were studied with FRET-based monitoring in a setup similar to that described above for the absorbance spectroscopy. In these experiments, the concentration of SbCPR-2DY and NADPH in the optical cell was equal to 5 and 100 µM, respectively, and the temperature was maintained at 5 °C. The composition of the other ingredients was the same as indicated above for the absorbance spectroscopy experiments. In these studies, the master channel of the MC2000-2 spectrometer was connected with a Vis-NIR 3 mm liquid light guide (Model 77635, Newport Corporation, Irvine, CA, USA) to the fluorescence window of the cell holder. The excitation light was provided with an M505F1 light-emitting diode (Thorlabs Inc., Newton, NJ, USA) emitting at 505 nm and functioning in a continuous wave mode. We used M617L3 light-emitting diode (Thorlabs Inc.) emitting at 617 nm as a light source in the experiments with direct excitation of the acceptor fluorophore. The fluorescence spectra were recorded in the range of 580–950 nm collected with the time intervals changing from 10 ms to 2 s per spectrum using custom data acquisition software.

### 2.5. Pressure Perturbation Experiments

Pressure-perturbation experiments were performed using a custom-built high-pressure optical cell [47] connected to a manual pressure generator (High Pressure Equipment, Erie, PA, USA) capable of generating a pressure of up to 6000 bar. The emission spectra were recorded with an MC2000-2 spectrometer (Ocean Optics) connected with a Vis-NIR 3 mm liquid light guide (Model 77635, Newport Corporation, Irvine, CA, USA) to the fluorescence window of the high-pressure cell. The spectra were recorded in the 580–900 nm region with a step of 1 nm. All spectra were corrected for the changes in protein concentration due to pressure-dependent compression of water as described earlier [48]. The excitation light was provided with an M505F1 light-emitting diode (Thorlabs Inc., Newton, NJ, USA) emitting at 505 nm in the continuous wave mode. The experiments were performed with 5 µM SbCPR-2DY at 25 °C in 20 mM Na-Hepes buffer. The experiments with the reduced SbCPR-2DY were carried out in the presence of an oxygen-scavenging system consisting of glucose oxidase (30 units/mL), 60 mM glucose, and 2000 units/mL catalase. The NADPH concentrations used in the experiments with the partially and fully reduced enzyme were equal to 5 and 100 µM, respectively. The experiments in the presence of 2′,5′-ADP were performed at 1 mM concentration of the latter.

### 2.6. Data Fitting

All data treatment and fitting, as well as the data acquisition in the absorbance and fluorescence spectroscopy experiments, were performed using our custom-designed SpectraLab software [48]. The latest version of the software package is freely available on the author’s website [49].

#### 2.6.1. Interpretation of the Results of Rapid Scanning Absorbance Spectroscopy

The series of spectra obtained in the rapid scanning absorbance stop-flow experiments were subjected to the Principal Component Analysis (PCA), a linear algebra method commonly used to reduce the dimensionality of large datasets [50]. It analyzes a set of M individual datasets (absorbance or fluorescence spectra in our case) of the dimensionality N (number data points in each spectrum). This dataset is used to construct the N × N covariance matrix, which is then transformed to find M-1 eigenvectors paired with M eigenvalues. The combination of each eigenvector with the corresponding set of eigenvalues is termed the Principal Component. The eigenvectors may be considered unified differences between the basis vector (the first spectrum in the series in our case) and other vectors (spectra) under analysis. The PCs are sorted based on their statistical significance. The first PC represents the most typical difference between the individual datasets (spectra), and the higher-order PCs contain the least significant deviations from the basis. This way, organizing information in PC allows reducing dimensionality without losing much information by discarding the components with low statistical significance. In practice, each dataset may be reconstituted with increasing accuracy by successive summarizing the basis vector and the eigenvectors multiplied by the respective eigenvalues. Thus, the set of eigenvalues deduced from the analysis of a spectral series reflects the changes in the amplitude of spectral alterations represented in the respective eigenvector, which might be considered a unified differential spectrum that characterizes the process under study. PCA is widely used for analyzing the results of rapid scanning kinetic experiments [51,52,53,54].

In our experiments, the first Principal Component yielded from this procedure typically covered over 98.5% of the total spectral changes. The time dependence of its eigenvalue was interpreted as reflecting the general kinetics of reduction. Approximation of these kinetics by a three-exponential equation was used to determine the kinetic constants of the individual phases of the reduction process.

#### 2.6.2. Interpretation of the Results of Fluorescence Spectroscopy

The quantitative interpretation of FRET results was based on the application of PCA to a series of emission spectra recorded in rapid kinetics or pressure perturbation experiments. Approximation of the first principal vector (>98% of the observed changes) with a combination of the prototypical spectra of emission of DY-520XL and DY731 normalized proportionally to their quantum yields (see Appendix A) allowed us to resolve the changes in the integral intensities of emission of each of the dyes and interpret them in terms of FRET efficiency. The quantum-yield-normalized intensities of the donor and acceptor fluorescence obtained in this way were used to determine the FRET efficiency according to the following equation:

(1) $$E=\left(I_{a}/\Phi_{a}\right)/\left(I_{a}/\Phi_{a}+I_{d}/\Phi_{d}\right)$$

where *I_a_* is the emission intensity of the acceptor, *I_d_* is the residual donor emission in the presence of acceptor, and Φ*_a_* and Φ*_d_* are the quantum yields of the fluorescence of the acceptor and the donor, respectively.

Distances between the donor and acceptor fluorophores (*R*) were calculated using the following equation:

(2) $$R=R_{0}\cdot \sqrt[{6}]{\left(E^{-1}-1\right)}$$

where *R*_0_ is the Förster distance calculated using the absorbance and emission spectra of the protein-bound donor and acceptor fluorophores. These calculations were performed using PhotoChemCad software [55], assuming the values of the orientation factor (*κ*) and the refractive index (*n*) to be equal to 0.667 and 1.4, respectively.

#### 2.6.3. Fitting of the Results of Pressure-Perturbation Experiments

The interpretation of the effect of pressure on protein equilibria in this article is based on the equation for the pressure dependence of the equilibrium constant ([56], Equation (1)):

(3) $$\partial \left(\ln K_{eq}\right)/\partial p=-\left(\Delta V^{0}\right)/RT$$

or in integral form, [57] (p. 212, Equtation (9)):

(4) Keq=Keq0·e−PΔV0/RT=e(P12−P)ΔV0/RT

where *K_eq_* is the equilibrium constant of the reaction at pressure *P*, *P*_½_ is the pressure at which *K_eq_* = 1 (“half pressure” of the conversion), Δ*V*^0^ is the standard molar reaction volume, and *K*^0^_*eq*_ is the equilibrium constant extrapolated to zero pressure, *K*^0^*_eq_* = *e*^*P*^_½_^Δ*V*^0^^/RT. For the equilibrium *A* ⇌ *B* and *K_eq_* = [*B*]/[*A*], Equation (4) may be transformed into the following relationship:

(5) [A]C0=11+Keq0·e−PΔV0/RT=11+e(P12−P)ΔV0/RT

where *C*_0_ = [*A*] + [*B*]. To determine the Δ*V*^0^ and *P*_½_ parameters from the experimental datasets describing pressure-induced changes in the amplitude of a signal derived from the fluorescence spectra (*A_p_*), this equation was complemented with the offset (*A*_0_) and scaling factor (*A_max_*) parameters and used in the following form:

(6) Ap=A0+Amax1+e(P12−P)ΔV0/RT

Prior to the analysis, all spectra were corrected for the compression of the solvent [48].

### 2.7. MALDI/TOF Analysis for the Fluorescence Dye Modified Peptides

Prior to MALDI/TOF analysis, the unmodified SbCPR-C596 and its adducts with monobromobimane (MBBr), DY-520XL and DY-731 (see Appendix B) were subjected to SDS-PAGE. After staining the gel slabs, their fragments containing the SbCPR protein band were isolated and subjected to in-gel trypsinolysis, following the established protocol [58]. The digested peptides were analyzed by MALDI/TOF MS. The peptide mass spectra were obtained using procedure and collection programs supplied by the manufacturer (Applied Biosystems, Waltham, MA, USA). The matrix, a-cyano-4-hydroxycinnamic acid, CHCA (Sigma-Aldrich, St. Louis, MO, USA), was prepared as a solution of 10 mg mL^−1^ in 50% water/acetonitrile with 0.1% TFA. The matrix solution was mixed 1:1 with the trypsin digest, applied to the sample plate, and dried. Spectra were collected using a 4800 MALDI TOF/TOF Analyzer (Applied Biosystems, Waltham, MA, USA), using the data collection programs in the positive mode for MS spectra.

## 3. Results

### 3.1. Selecting the Attachment Points for the Fluorophores and Construction of the Cysteine-Depleted Variant of SbCPR

To explore the conformational dynamics of SbCPR that accompanies the process of its NADPH-dependent reduction and probe the thermodynamic parameters of the respective conformational transitions with pressure-perturbation spectroscopy, we sought to introduce a FRET donor/acceptor pair into the FAD and FMN domains of the enzyme. The background for selecting DY-520XL and DY-731 maleimides as thiol-reactive fluorophores for these experiments is described in Appendix A. There we also provide characterizations of the photochemical properties of this donor/acceptor pair and its incorporation into SbCPR.

**Figure 1:**
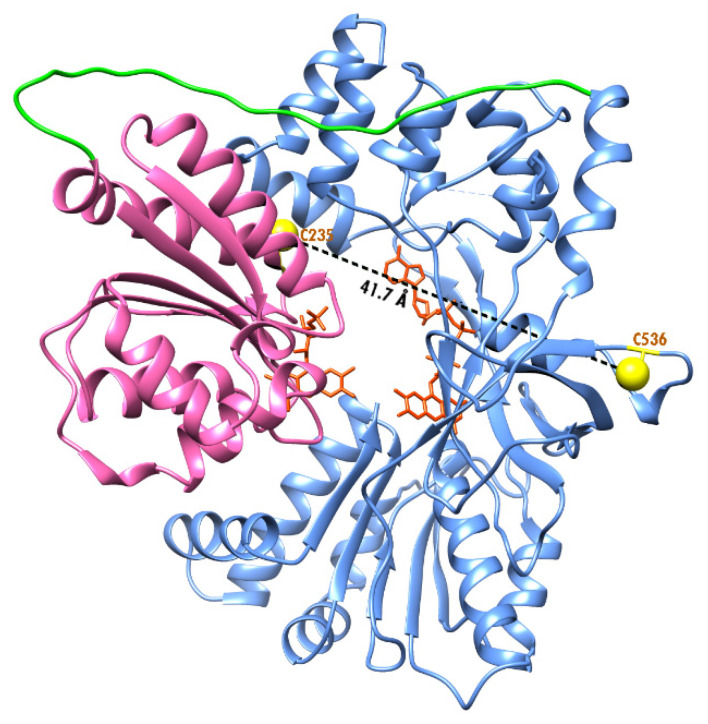
Structural model of SbCPR based on a combination of the resolved X-ray structure of the FAD domain (PDB ID: 7SUX) with the model of FMN domain and the connecting loop built with AlphaFold. The two modification-accessible cysteine residues are shown as yellow spheres. FMN and FAD domains are pink and light blue, respectively, and the connecting loop is green. FAD and FMN molecules are shown as orange stick models. The N-terminal transmembrane helix of the enzyme is not shown.

To minimize the perturbations of the protein structure by mutagenesis needed for the site-directed incorporation of the thiol-reactive probes, we elected to employ some of the native cysteine residues of the protein for its modification. SbCPR contains nine cysteine residues—Cys-235, Cys-306, Cys-325, Cys-352, Cys-503, Cys-520, Cys-536, Cys-596 and Cys659. According to the analysis of the structure of the FAD domain [7] and the homology model of the full-length enzyme, the residues Cys-306, Cys-325, Cys-352, Cys-503, Cys-520, and Cys-659 are buried inside the structure and barely accessible. Cys-235, the only cysteine residue located in the FMN domain, and Cys-536, located on the surface of the FAD/NADPH-binding domain, appear to be easily accessible for modification. Another potentially accessible cysteine in the FAD domain is Cys-596, which is more buried than Cys-536. Thus, we expect to encounter two–three modification-accessible cysteine residues in SbCPR, Cys-235, Cys-536, and Cys-596. Of those three residues, the Cys-235 and Cys-536 provide an optimal combination for positioning the donor/acceptor pair. The position of these residues is indicated in Figure 1, where we show a model of SbCPR based on a combination of the resolved X-ray structure of its FAD domain (PDB ID: 7SUX) [7] with the model of the FMN domain and the connecting loop built with AlphaFold, a machine-learning-based protein structure prediction tool [59]. The model was created by replacing the FAD domain in the AlphaFold-generated model of SbCPR with its X-ray-resolved structure starting at Val-310 residue. To this aim, the AlfaFold-built model of SbCPR and the X-ray structure of the FAD domain were aligned upfront using the MatchMaker tool of the UCSF Chimera software [60].

As described in Appendix B, studying the accessibility of SbCPR cysteines for modification with monobromobimane (MBBr), we demonstrated that the enzyme contains three cysteine residues easily accessible for modification with a thiol-reactive probe. Employing MALDI-TOF mass spectroscopy for probing the points of attachment of the fluorophores, we confirmed the above conclusion that Cys-235, Cys-536, and Cys-596 (listed in the order of decreasing accessibility) are the most modification-accessible cysteine residues in the protein.

To limit the SbCPR modification by thiol-reactive probes to a pair of most readily available cysteine residues, Cys-235 and Cys-536, we removed Cys-596 by mutating it into a serine residue. The resulting C596S variant, with only eight cysteine residues per enzyme molecule, can be readily expressed in *E. coli* and purified with a yield similar to that characteristic of the wild-type SbCPR. The rate of its turnover in the reduction of cytochrome c was also nearly identical to that exhibited by the wild-type enzyme. The C596S variant was therefore used in all experiments described in this article. It is from now on referred to as SbCPR to simplify the narrative.

The sequential modification of the C596S variant of SbCPR with DY520-XL and DY-731 (see Section 2.3) resulted in the protein that contains 0.7–1 molar equivalent of each dye per protein molecule and did not result in any considerable protein precipitation. The modified C596S variant enzyme, which we designate hereafter as SbCPR-2DY, was active in the cytochrome c reduction with the turnover number of 2267 min^−1^, which is comparable with that observed in the wild-type SbCPR. Our MALDI-TOF MS analysis described in Appendix B suggests that the sequential labeling procedure results in the protein where the DY-520-XL probe is prevalently attached to Cys-235, while DY-731 fluorophore is by preference located at Cys-536.

### 3.2. Kinetics of the NADPH-Dependent Reduction of SbCPR

In order to explore the time frame of the individual steps of the NADPH-dependent reduction of SbCPR, we examined its kinetics by rapid-scanning stop-flow absorbance spectroscopy. The reduction process was followed by monitoring the changes in absorbance in the 350–700 nm region.

The oxidized state of CPR flavins has two major absorbance bands centered at 380 and 456 nm, respectively. In the neutral (blue) and anionic (red) flavin semiquinones, the amplitude of the band at 456 nm is dramatically decreased. Furthermore, the neutral semiquinone state is distinguished by a broad band centered around 595 nm, which is lacking in the anionic semiquinone. In addition to the complete lack of the 456 and 595 nm bands, the two-electron reduced (hydroquinone) state differs from the semiquinones by decreased absorbance at 380 nm. Therefore, the process of the transition of the enzyme through the stages depicted in Scheme 1 can be followed by analyzing the changes in absorbance at 380, 456, and 595 nm. A decrease in the absorbance at 456 nm reflects the overall reduction process. The changes in the amplitude of the absorbance band at 595 nm reflect the initial appearance and further evanescence of the neutral flavin semiquinones (FADH^•^, FMNH^•^). The formation of the two-electron reduced flavin hydroquinones (FADH_2_ and FMNH_2_) can be judged from a decrease in absorbance at 380 nm.

**Figure 2:**
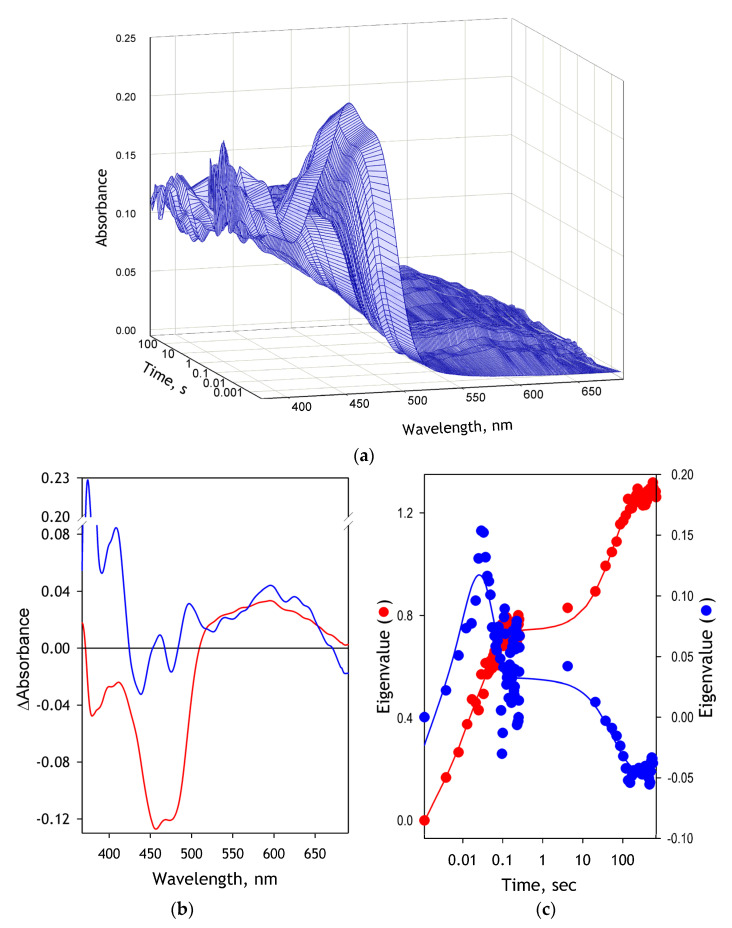
Changes in the spectra of absorbance of SbCPR in the process of its NADPH-dependent reduction. Panel (**a**) exemplifies a series of absorbance spectra recorded in a rapid-scanning stop-flow experiment. Panel (**b**) shows the spectra (eigenvectors) of the first (red) and second (blue) Principal Components obtained with PCA of the above dataset, and panel (**c**) represents the respective sets of eigenvalues plotted against time in semi-logarithmic coordinates. Solid lines in this panel correspond to the approximations of the kinetic curves with the three-exponential equation.

Results of our stop-flow experiments are presented in Table 1 and Figure 2. A series of spectra recorded during the reduction process is exemplified in a 3D plot, shown in Figure 2a. Principal Component Analysis (PCA) of this dataset results in the first and the second principal components (PCs) covering 98.6 and 0.6% of the total changes, respectively. The respective eigenvectors and the sets of eigenvalues are shown in Figure 2b,c. As seen from these plots, the spectra of both PC (their eigenvectors) reveal the features characteristic of the changes in flavins’ redox state. The first PC possesses minima at the positions of both absorbance bands of oxidized flavins (380 and 456 nm) and a maximum at 595 nm that corresponds to the absorbance band of the blue semiquinone. While this PC characterizes the overall kinetics of reduction, the second PC, which features the bands at 375 and 595 nm, reflects the difference in the spectral changes between the individual reduction stages (Scheme 1).

For both principal components, the kinetics of the changes in eigenvalues can be adequately approximated with the three-exponential equation (Figure 2c). These approximations yield the sets of kinetic constants that closely match each other. Conversely, an obvious distinction between the two components in the signs and the amplitudes of the individual exponential terms reveals a difference in the spectral signatures of the separate phases of the reduction process. The kinetic constants of the individual phases determined from the fitting of the kinetics of changes in the first eigenvalue to the three-exponential equation and averaged over 12 kinetic runs were found equal to 168 ± 29, 26.9 ± 9.8, and 0.021 ± 0.010 s^−1^. These values can also be found in Table 1, along with those obtained in the kinetic experiments with FRET-based detection (see below).

**Figure 3:**
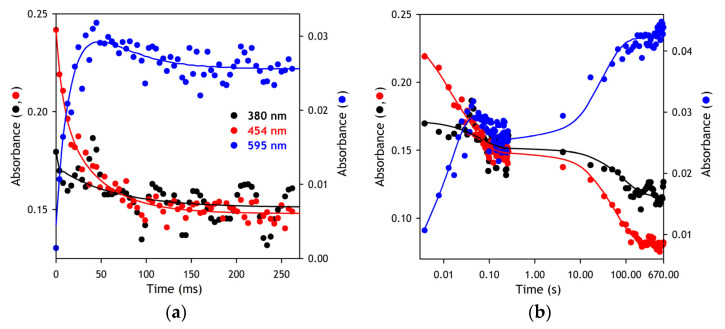
Changes in absorbance of SbCPR at 380, 454, and 595 nm in the process of its NADPH-dependent reduction. Panel (**a**) shows the initial parts of the curves in linear coordinates. In panel (**b**), the same datasets are plotted versus the logarithm of time in the entire time range of the experiment. Solid lines correspond to the approximations of the kinetic curves with the three-exponential equation.

Although the use of PCA provides a potent means for global analysis and allows for the accurate determination of the kinetic constants of the individual phases, the identification of the stages of the reduction process that correspond to each of the three exponential terms might be better achieved through the analysis of the absorbance changes at the three representative wavelengths (380, 456 and 595 nm). The respective kinetic curves are exemplified in Figure 3. As seen from this figure, even the first spectrum taken after the mixing is characterized by a substantial increase in absorbance at 595 nm, which is indicative of the formation of the neutral semiquinones. The increase in absorbance at this wavelength continues for approximately 20 ms. It is followed by a partial reversal within the subsequent 200–300 ms. These two initial phases are associated with a profound decrease in the absorbance at 456 nm accompanied by a much less significant attenuation in the optical density at 380 nm.

According to our interpretation, the changes in SbCPR absorbance during the first 500 ms of the reduction process (Figure 3a) suggest that the first resolved kinetic phase corresponds to the inter-flavin electron transfer event (stage (2) in Scheme 1). The subsequent slower phase may be identified to the stage (3), where the transient equilibrium between different two-electron reduced forms of the enzyme is established. These two rapid phases are followed by a very slow further decrease in absorbances at both 456 and 380 nm, accompanied by an increase in the amplitude of the 595 nm band. According to our interpretation, this stage corresponds to the reduction of some part of the enzyme pool to the four-electron reduced state followed by a comproportionation reaction between two- and four-electron reduced SbCPR molecules [61] that leads to the three-electron reduced enzyme (FADH_2_, FMNH^•^), which appears to be the predominating end-state of the reduction process.

### 3.3. Conformational Transitions in SbCPR Studied with FRET and Stop-Flow Spectroscopy

**Figure 4:**
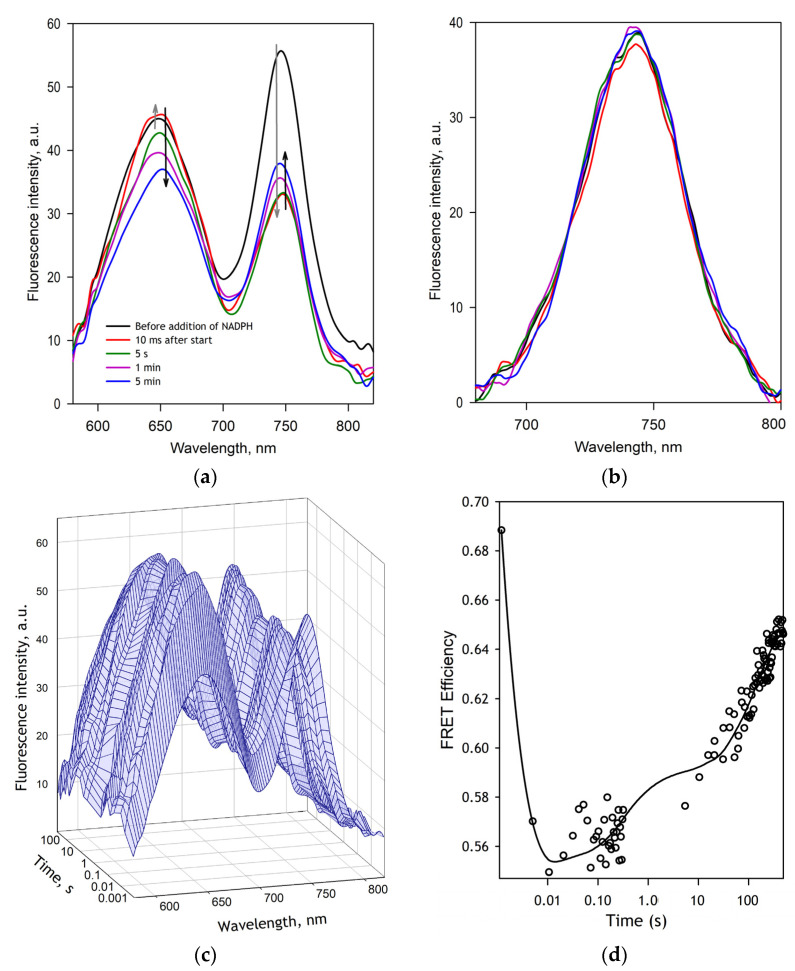
Changes in the fluorescence of SbCPR-2DY in the process of its NADPH-dependent reduction studied by rapid-scanning stop-flow spectroscopy. Panel (**a**) shows a representative set of spectra of fluorescence with excitation at 505 nm registered at different time points. Here, the gray arrows indicate the direction of the changes during the initial 100 ms of the process. Black arrows indicate the direction of the changes during the latter phase of reduction. A series of spectra of fluorescence recorded at the same time points with the direct excitation of the acceptor at 617 nm is shown in panel (**b**). Panel (**c**) illustrates the spectral changes during the reduction as a 3D plot of fluorescence spectra (excitation at 505 nm) normalized on the integral fluorescence intensity. A kinetic curve of the changes in FRET efficiency during the reduction is shown in panel (**d**) in semi-logarithmic coordinates. The solid line shown in this plot corresponds to the approximation of this data set by a three-exponential equation.

To explore the fluctuations of the SbCPR conformational landscape during the redox cycling of the enzyme, we studied the kinetics of changes in FRET in SbCPR-2DY observed in the process of its anaerobic reduction with NADPH with stop-flow technique combined rapid scanning fluorescence spectroscopy. A series of fluorescence spectra taken during the reduction process is exemplified in Figure 4a. As seen from this figure, the addition of NADPH results in a profound drop in the fluorescence of DY731, the FRET acceptor. This extremely rapid decrease is followed by a slow partial reversal associated with a considerable increase in the fluorescence of DY520XL, the FRET donor.

To probe whether the observed changes in the fluorescence of the acceptor reflect the changes in the FRET efficiency and are not associated with the possible alteration of the fluorescence of the acceptor per se, we performed stop-flow experiments with the direct excitation of the acceptor fluorophore at 617 nm. A series of spectra of DY731 fluorescence monitored during the reduction process in this setup is exemplified in Figure 4b. As seen from this plot, the intensity of DY731 fluorescence exhibits no noticeable changes during the reduction. Therefore, the spectral changes observed in the experiments with excitation at 505 nm may be unequivocally attributed to the changes in FRET efficiency.

To assess the FRET efficiency in these experiments and estimate its changes during the reduction, we normalized the series of spectra recorded in the stop-flow experiments on the total intensity of fluorescence of both fluorophores corrected according to their quantum yields (see Appendix A). A series of spectra normalized in this way is shown as a 3D plot in Figure 4c. As described in Materials and Methods, the efficiency of FRET may be directly assessed by calculating the relative intensity of fluorescence of the acceptor normalized in this way (see Equation (1) in Section 2.6.2).

Figure 4d exemplifies a kinetic curve of the changes in FRET efficiency during SbCPR reduction. As seen from this figure, the addition of NADPH to the enzyme results in a very rapid drop in FRET efficiency, followed by a slow two-exponential increase. At the end of this process, the FRET efficiency returns to a level close to that characteristic of the oxidized enzyme. Similar to the kinetics of reduction per se, these kinetic curves may be approximated with a three-exponential equation. The kinetic constants and the phase amplitudes derived from these approximations are found in Table 1.

As seen from Table 1, where we compare the kinetic parameters of the SbCPR reduction with those characterizing the kinetics of changes in FRET, the two processes exhibit similar values of the rate constants of all three phases. Thus, the rapid initial decrease in FRET intensity may be identified as reflecting the sequence of events resulting in the conversion of the oxidized enzyme (FAD, FMN), first to (FADH_2_, FMN) then to the double-semiquinone state (FADH^•^, FMNH^•^) (stages 1–2 in Scheme 1). A decrease in FRET efficiency in this phase suggests an increase in the average inter-probe distance by ~4 Å. This increase indicates that the formation of the disemiquinone state of the enzyme prompts it to acquire a more open conformation. However, this opening is reversed in the following stages of reduction so that the inner-probe distance in the final, apparent three-electron reduced state approaches that characteristic to the oxidized enzyme.

### 3.4. Pressure-Perturbation Studies of the Equilibrium between Open and Closed States in SbCPR

The FRET-based detection of the conformational rearrangements in SbCPR established in the above-described experiments opens a gate to apply pressure-perturbation spectroscopy for exploring the conformational landscape of the enzyme and its alterations in redox cycling. The pressure perturbation approach offers unique means for probing the changes in protein–solvent interactions and determining the position of conformational equilibria in different states of the enzyme. Thus, we subjected SbCPR-2DY to a cycle of studies of the effect of pressure on the fluorescence of the donor–acceptor pair. These experiments were performed with the oxidized enzyme, its complex with 2′,5′-ADP (as an NADPH analog), the two-electron reduced enzyme in the presence of equimolar NADPH, and its final, apparently three-electron reduced state at a 20-fold excess of NADPH.

**Figure 5:**
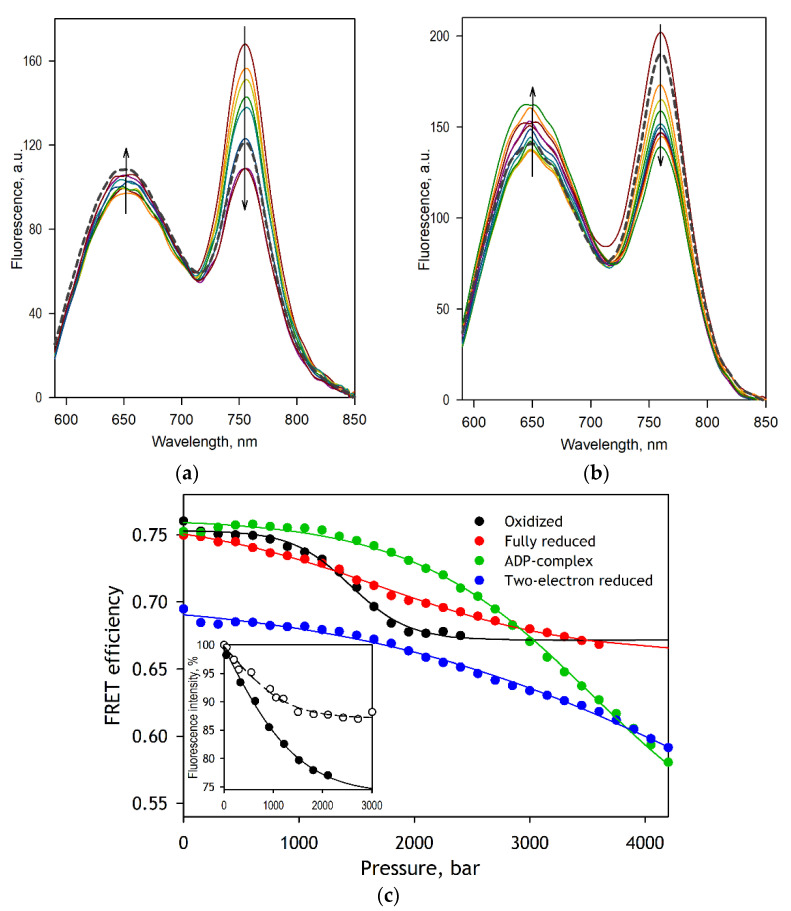
Pressure-induced changes in the spectra of fluorescence of SbCPR-2DY. Panels (**a**,**b**) show the series of spectra recorded at increasing pressure with the oxidized (**a**) and reduced (**b**) enzyme. The spectra shown in solid lines were recorded at 1, 300, 600, 900, 1200, 1500, 1800, 2100, and 2400 bar. In the case of the reduced enzyme, this set is complemented with the spectra recorded at 3000, 3600, and 4200 bar. The spectra shown in thick gray dashed lines were recorded after decompression to the ambient pressure. The pressure dependencies of FRET efficiency obtained with four different enzyme states are exemplified in the main plot of the panel (**c**). Here, the solid lines show the approximations of the datasets with Equation (6). The inset in this panel exemplifies the pressure dependencies of the relative fluorescence intensity of the double-labeled enzyme with excitation at 617 nm (filled circles, solid line) and single-labeled SbCPR-DY520XL with excitation at 505 nm (open circles, dashed line). Lines show the approximations of the datasets with Equation (6).

The results of our pressure-perturbation experiments are illustrated in Figure 5, where panels (a,b) exemplify the series of spectra recorded versus increasing pressure with the oxidized and reduced enzyme, respectively. As seen from these plots, rising pressure results in a remarkable increase in the emission band of the acceptor accompanied by a decrease in the donor fluorescence. A similar behavior was also observed with ADP-bound SbCPR and its two-electron reduced state.

Notably, the changes observed with either the completely or partially reduced enzyme were entirely reversible at decompression (see Figure 5b). In contrast, in the oxidized state of either the ligand-free or ADP-bound enzyme, full reversibility was observed only when the enzyme was decompressed from pressures below 1.5 kbar. At higher pressures, the reversibility was only partial (see Figure 5a). Furthermore, the prolonged incubation of the oxidized SbCPR at pressures above 2.5 kbar resulted in an ample time-dependent irreversible decrease in the fluorescence of the acceptor along with increased emission from the donor (data not shown). This observation suggests the slow pressure-induced denaturation of the oxidized enzyme at pressures >2.5 kbar. In contrast, no pressure-induced denaturation was observed with either partially or completely reduced enzymes at pressures as high as 4.2 kbar.

To probe if the pressure effects on the fluorescence of the donor–acceptor pair may be, at least in part, caused by pressure dependence of the quantum yield of each of the probes taken alone, we studied the effect of pressure on the fluorescence of DY-520XL in the single-labeled SbCPR-DY520XL and the fluorescence of DY-731 in SbCPR-2DY subjected to direct excitation at 617 nm. The obtained pressure dependencies are shown in the inset to Figure 5c. This plot shows that both dyes exhibit pressure-dependent quenching of fluorescence, which is better pronounced with DY-731. Thus, the opposite directions of the changes in the intensity of fluorescence of the donor and acceptor fluorophores in the double-labeled enzyme with excitation at the donor band (at 505 nm) suggest a pressure-induced decrease in FRET efficiency, and, therefore, an increase in the distance between the probes at increasing pressure.

The effect of pressure on the intensity of donor fluorescence must equally influence the amplitudes of the bands of both donor and acceptor fluorophores and, therefore, do not affect the calculations of FRET efficiency according to Equation (1). In contrast, the changes in the quantum yield of DY-731 with pressure have to be taken into account in our calculations. To this end, we fitted the pressure dependence of the relative intensity of fluorescence of DY-731 by Equation (6) (Figure 5c, inset) and used this fitting curve to normalize the amplitude of the fluorescence band of the acceptor. The resulting pressure dependencies of FRET efficiency for the four enzyme states are shown in Figure 5c main panel. The respective parameters of pressure-induced transitions are summarized in Table 2.

Although pressure increase elicited a decrease in FRET efficiency in all four cases, both the parameters of the pressure-induced transitions and the estimated changes in the inter-probe distance reveal a dramatic difference between the four states of the enzyme. While the distance between the probes in the low-pressure end state (the closed conformation) did not differ considerably between the four states, the inter-probe distances in the high-pressure end state (the open conformation) of the ADP-bound enzyme and its two-electron reduced form were significantly longer than those characteristic to the oxidized and completely reduced forms. While in the latter two cases, the change in the distance upon the transition from more closed to more open conformation was estimated to be 2–4 Å, the opening of the ADP-bound and two-electron reduced enzyme increases the inter-probe difference by 8–10 Å.

A contrasting difference between the oxidized enzyme and its other three states was also observed in the Δ*V*^0^ of the opening transition. If the decrease in the system volume upon its pressure-induced transition was as large as 88 mL/mol for the oxidized enzyme, the binding of 2′,5′-ADP and the reduction of the flavins decreased this change to −33 and −18 mL/mol, respectively. This observation suggests that the conformational change in SbCPR resulting from its interaction with the nucleotide cofactor and further reduction promotes additional protein solvation that minimizes the changes in protein interactions with solvent necessary for acquiring its open conformation.

Importantly, our analysis of the *K_*eq*_* of the pressure-dependent conformational equilibrium suggests that, although the closed conformation predominates in all four enzyme states at ambient pressure, the formation of the disemiquinone state results in a ~10-fold increase in the population of the open state. Interestingly, the further reduction of the enzyme shifts the equilibrium towards the open state even more. However, the “degree of opening” observed in the final, the apparent three-electron reduced state is much lower than in the ADP-bound and two-electron reduced states.

## 4. Discussion

Incorporating DY520-XL and DY731 fluorescence dyes into the FAD and FMN domains of CPR from *Sorghum bicolor* provided means for the direct observation of conformational rearrangements during the redox cycling of the enzyme. A close match of the kinetic constant derived from the absorbance and fluorescence spectroscopy assays in rapid scanning stop-flow experiments allowed us to track the changes in the average inter-probe distance during the flavoprotein reduction process. Furthermore, applying pressure-perturbation spectroscopy for portraying the conformational landscape of the enzyme, we were able to determine the positions of equilibrium between its open and closed conformations at different points of the electron-transfer pathway. These studies revealed the presence of several open protein sub-states that differ in the protein solvation pattern. Through unveiling the functional choreography of plant CPRs, our study provides the essential information for rational engineering these pivotal enzymes of the monolignol biosynthetic pathway.

The results of our rapid kinetics experiments suggest that the binding of NADPH to the enzyme is exceptionally fast and takes place during the dead time of the stop-flow device (~2 ms). Similar to that reported for human CPR [62], the reduction of SbCPR by NADPH occurs without the intermittent occurrence of the detectable charge-transfer species. The first resolved kinetic phase is the formation of the blue (neutral) disemiquinone resulting from the interdomain electron transfer between FADH_2_ and FMN immediately coupled to the charge-transfer step. However, the rate constant of this process (around 200 s^−1^ at 5 °C, see Table 1) is an order of magnitude higher than the values reported by Gutierez et al. for human CPR (20 s^−1^ at 25 °C) [62] and by Oprian and Coon for rabbit CPR (28 s^−1^ at unspecified temperature) [63]. Thus, the interdomain electron transfer in SbCPR occurs much faster than in the mammalian enzymes. This observation is consistent with the higher flexibility of the interdomain loop in plant CPRs as compared to the mammalian orthologs, which is suggested by their X-ray structures [7,11].

The second kinetic step presumably corresponds to establishing a transient equilibrium of the red and blue disemiquinones with the other two-electron reduced states, (FAD, FMNH_2_) and (FADH_2_, FMN). It has a rate constant of around 20 s^−1^ (Table 1), which is also much higher than those reported for the human (3.7 s^−1^ [62]) and the rabbit (5.4 s^−1^ [63]) enzymes. Notably, the amplitude of the 595 nm band and, respectively, the fractional content of the blue semiquinone species at the end of this phase is considerably higher in SbCPR than that observed with the mammalian reductases.

Interestingly, the kinetics of the reduction of SbCPR reveals a noticeable difference with that reported for another plant CPR, ATR2 enzyme from *Arabidopsis thaliana*. Studying this enzyme, Whitelaw and co-authors reported a resolution of the hydride transfer step (425 s^−1^ at 6 °C) from the interdomain electron transfer stage (49 s^−1^) [28]. Thus, according to our results, both of these electron transfer events appear to be considerably faster in SbCPR than in ATR2. Furthermore, the fractional content of the blue semiquinone state in the transient equilibrium mixture established after the second phase of reduction is markedly higher in SbCPR than in ATR2, similar to what is observed in the comparison of SbCPR with the mammalian reductases.

The third, extremely slow, kinetic phase of the reduction corresponds to a partial transition of the enzyme to the four-electron reduced state followed by a comproportionation between the two- and four-electron reduced molecules leading to the three-electron reduced enzyme ((FADH_2_, FADH^•^) ⇌ (FMNH^•^, FMNH_2_)), the apparent final state. This incomplete reducibility of CPR and the formation of a three-electron reduced state through a comproportionation reaction has already been reported for mammalian reductases [64] and the flavoprotein domain of the bacterial P450BM-3 [61].

The changes in FRET efficiency in SbCPR-2DY during its NADPH-dependent reduction are in good agreement with the kinetics of changes in the redox state of the enzyme. The kinetic curves registered in our fluorescence stop-flow experiments obey three-exponential kinetics with the rate constants closely similar to those obtained with absorbance spectroscopy. According to our results, the first resolved phase of reduction that leads to the disemiquinone state is associated with an increase in the averaged inter-probe distance by 4.1 Å. However, the subsequent equilibration between several two-electron reduced states and the further reduction of the enzyme results in the opposite direction of the changes so that the averaged distance observed in the three-electron reduced enzyme is only 1.7 Å larger than in the oxidized enzyme (Table 1).

These results agree with multiple previous reports that demonstrated a transition from the closed conformation to a more open state upon the interdomain electron transfer event and the formation of the disemiquinone state (see [27] for a review). Furthermore, there are strong indications of the predominance of the closed conformations in the three- and four-electron reduced enzyme [20,21,23,26], which is also consistent with our results.

It must be noted that the inter-probe distances estimated in our FRET kinetics experiments do not reflect the distances characteristic of any discrete protein conformations. They rather represent the averages over multiple conformational states, and their changes reflect the redox-state-dependent displacement of equilibrium between these various conformations. To explore better the conformational landscape of SbCPR and characterize its changes in the redox cycling of the enzyme, we used hydrostatic pressure as a tool for displacing the protein equilibria. When combined with such tools for detecting protein structural rearrangements as FRET, the pressure perturbation strategy allows determining the positions of its conformational equilibria in different redox states, assessing the changes in the protein interactions with solvent in its redox cycling, and estimating the inter-probe distances characteristic to the end-states of its conformational breathing.

According to our results, in all four studied states of the enzyme—oxidized ligand-free, oxidized ADP-bound, two-electron, and four-electron reduced—the enzyme exists in the equilibrium between its more closed and more open states. The inter-probe distance in the closed state is not affected by either reduction or the interactions with 2′,5′-ADP (the analog of the nucleotide cofactor). It is estimated to be around 38–40 Å in all four states of the enzyme (Table 2). In contrast, the preferential conformation of the open state exhibits a pronounced change during the redox cycling. While the changes in the inter-probe distance associated with the conformational breathing of the oxidized enzyme are as small as 2 Å, the binding of ADP to SbCPR and its subsequent two-electron reduction increases the distance between the probes in the open conformation to 46–50 Å (Table 2). In agreement with the previous observations [20,21,23,26], the further reduction of the enzyme partially reverts this change and decreases the amplitude of the conformational motion to 4 Å.

A unique feature of the pressure-perturbation approach is its ability to reveal conformational equilibria’s endpoints. Suppose that the inter-probe distances observed at any particular pressure represent weighted averages over a population of molecules existing in equilibrium between the low-pressure and pressure-promoted conformational states. Hence, the inter-probe distances estimated by extrapolating pressure dependencies to infinitely high and infinitely low pressures correspond to those characterizing the definite states (or ensembles of states with pressure-insensitive interconversion) representing the endpoints of conformational equilibrium. Therefore, comparing the end states of pressure-dependent transitions of SbCPR at different points of its redox cycle allows probing if the interactions of the enzyme with the nucleotide cofactor or reduction of its flavins results in an emergence of a new conformational state not present in the oxidized enzyme. The remarkable difference of the inter-probe-distance in the pressure-promoted end-state of the oxidized enzyme with those observed in SbCPR-ADP complex and two-electron reduced enzyme suggests that the binding of the nucleotide cofactor is a necessary prerequisite for the wide opening of the enzyme. This inference is consistent with a dramatic difference of the Δ*V*^0^ characteristic to the oxidized enzyme (89 mL/mol) with the Δ*V*^0^ values exhibited by SbCPR in all other probed states. According to this analysis, the nature of the low-amplitude conformational breathing observed in oxidized SbCPR is entirely different from the transitions between the closed and widely open conformations, which become possible only after binding the nucleotide cofactor.

However, it should be taken into account that the changes in the distance between the two protein-incorporated probes monitored in our experiments do not reflect the complete picture of the structural rearrangements, which may involve complex rotational and translational motions of different parts of the protein molecule. Therefore, the inter-probe distance may not be considered a full-fledged measure of the “degree of openness” of the enzyme molecule. Consequently, the actual variations in the accessibility of the FMN domain for interactions with the heme protein acceptor may not be exactly proportional to the variations in the inter-probe distances detected in our experiments.

The most notable and nontrivial conclusion derived from the fitting of the pressure dependencies of the FRET efficiency to Equation (6) is that the closed conformation of the enzyme heavily predominates in all four studied states of the enzyme at ambient pressure. According to the constant of equilibrium determined for the oxidized enzyme, the fraction of the closed form accounts for 99.6% of its total content. Despite a significant increase in the amplitude of the conformational breathing upon the binding of the nucleotide cofactor, its effect on the position of the conformation equilibrium is insignificant (99% of the closed state at 1 bar). However, the reduction of the enzyme with the formation of the disemiquinone displaces the equilibrium appreciably, and the fraction of the closed state decreases to 96%. Surprisingly, despite decreasing the amplitude of motions, the further reduction of SbCPR provokes the further opening of the enzyme so that the fraction of its closed form decreases to 89%.

It has to be noted that the high abundance of the closed conformation of the enzyme in our experiments is furthered by the low ionic strength of the buffer used in our studies (20 mM Na-HEPES, *I* = 11.6 mM). The conformational equilibrium in CPR enzymes is known to be critically affected by ionic strength, and the abundance of open conformation is considerably increased at high salt concentrations [19,24,25]. This strong ionic strength dependence is caused by the predominant role of charge pairing contacts in the inter-domain interactions [12,65]. The use of the low-ionic-strength media in our experiments was dictated by an objective to accurately determine FRET efficiency in the low-pressure (closed) end-state by displacing the *P*_½_ of the opening transition to higher pressures. It is worth noting that the cytoplasmic ionic strength in cells of plants grown at normal soil salinity varies within 100–200 mM limits [66], which is much higher than the ionic strength of our buffer (11.6 mM). Therefore, the actual position of the SbCPR conformational equilibrium in vivo must be more shifted towards the open state than observed in our experiments.

Another remarkable observation is a dramatic change in the Δ*V*^0^ of the opening transition upon the binding of 2′,5′-ADP and the enzyme reduction. If in the oxidized SbCPR the volume change is as large as −88 mL/mol, the binding of the nucleotide cofactor decreases it (by absolute value) to −33 mL/mol. The subsequent electron transfer to the flavins and formation of the disemiquinone state further decreases Δ*V*^0^ to −18 mL/mol. This observation suggests that the binding of the nucleotide cofactor and two-electron reduction results in some additional hydration of the enzyme, which decreases the changes in protein interactions with solvent necessary for the transition to the open conformation. Suppose we hypothesize that the predominant part of the volume change is originated from the electrostriction of water on the newly opened charges after breaking salt bridges. In that case, we can assume that the opening of the oxidized enzyme involves the dissociation of four salt links. That is calculated from the assumption that the solvation of a single-charged ion incurs the volume change of −10 mL/mol [31]. In contrast, there is only one salt bridge to break for the opening transition of the two-electron reduced enzyme (Δ*V*^0^ = −18 mL/mol).

The analysis of the recently resolved X-ray structure of CPR from the *Candida tropicalis* yeast identified four salt-bridges connecting the FAD and FMN domains in the closed conformation of the enzyme [12]. Comparing this structure with our structural model of SbCPR, we found that three of these charge pairs—D125/R515, E157/K669, and E193/R367—are retained in the sorghum enzyme, where they correspond to the pairs D168/R544, E203/K693, and D238/K414. The fourth one (K57/D338) has no analog in SbCPR despite the conservation of K57 residue, which corresponds to K96 in SbCPR. However, instead, this residue may be a part of a switching salt-link fork between D260 in the connecting loop and K96 and K126 in the FMN domain. Dissociation or switching these salt links may affect the flexibility of the connecting loop and thus modulate the interdomain interactions. Hypothetically, the dissociation of these salt bridges may be involved in the pressure-induced opening of the ligand-free oxidized enzyme. A decrease in the Δ*V*^0^ of the opening transition upon the binding of the nucleotide cofactor and NADPH-dependent reduction may indicate that the interactions of the enzyme with 2′5′-ADP or NADPH promote the dissociation of some of these salt links. Further investigation of this system of molecular tethering and its linkage to the enzyme redox-state by a combination of site-directed mutagenesis and pressure-perturbation FRET spectroscopy may give more insight into the mechanisms controlling the inter-domain interactions in SbCPR.

## 5. Conclusions

Combining FRET-based detections of conformational motions with pressure-perturbation and rapid scanning stop-flow spectroscopy, we were able to portray the conformational landscape of the enzyme and characterize its changes in the process of enzyme redox cycling. Our results suggest the presence of several open conformational sub-states differing in the opening transition volume change (Δ*V*^0^). Although the closed conformation always predominates in the conformational landscape, the population of the open conformations increases by order of magnitude upon the two-electron reduction and the formation of the disemiquinone state of the enzyme. In addition to elucidating the functional choreography of plant CPRs, our study demonstrates the high exploratory potential of a combination of the pressure-perturbation approach with the FRET-based monitoring of protein conformational rearrangements.

## Figures and Tables

**Table 1:** Parameters of the SbCPR reduction as determined with absorbance and fluorescence stop-flow spectroscopy *.

Phase of the Reduction Process	Apparent End State	Absorbance Spectroscopy	Fluorescence Spectroscopy		
		*k*_obs_, s^−1^	*k*_obs_, s^−1^	FRET Efficiency in the End State	Apparent Inter-Probe Distance in the End State, Å
(initial state)	$\begin{array}{c} \mathrm{FAD}\\ \mathrm{FMN} \end{array}$			0.73 ± 0.03	41.0 ± 1.0
1	$\begin{array}{c} {\mathrm{FADH}}^{•}\\ {\mathrm{FMNH}}^{•} \end{array}$	168 ± 29	230 ± 71 (0.089)	0.60 ± 0.03	45.1 ± 1.0
2	$\left(\begin{array}{c} {\mathrm{FADH}}_{2}\\ \mathrm{FMN}\; \end{array}↔\begin{array}{c} {\mathrm{FADH}}^{•}\\ {\mathrm{FMNH}}^{•} \end{array}↔\begin{array}{c} \mathrm{FAD}\\ {\mathrm{FMNH}}_{2} \end{array}\right)$	26.9 ± 9.8	17.3 ± 12.7 (0.171)	0.63 ± 0.02	44.2 ± 0.6
3	$\begin{array}{c} {\mathrm{FADH}}^{•}\\ {\mathrm{FMNH}}_{2} \end{array}↔\begin{array}{c} {\mathrm{FADH}}_{2}\\ {\mathrm{FMNH}}^{•} \end{array}$	0.021 ± 0.010	0.024 ± 0.012 (0.686)	0.68 ± 0.01	42.7 ± 0.4

* The values given in the table represent the averages of the results of 3–6 individual experiments. The “±” values show the confidence interval calculated for *p* = 0.05. The values given in parentheses represent the results of the Student’s t-test for the hypothesis of equality of the *k*_obs_ values deduced from the absorbance experiments to those obtained in the FRET studies.

**Table 2:** Parameters of the pressure-induced transition in SbCPR-2DY monitored by changes in FRET efficiency *.

State of the Enzyme	*P*_½_, bar	Δ*V*^0^, mL/mol	*K* *_eq_*	Δ*G*°, kJ/mol	Low-Pressure End State (Closed)		High-Pressure End State (Open)	
					FRET Efficiency	Distance, Å	FRET Efficiency	Distance, Å
Oxidized	1719 ± 235	−87.8 ± 20.8	0.004 ± 0.004	6.47 ± 1.26	0.76 ± 0.01	38.1 ± 0.3	0.71 ± 0.04	40.1 ± 0.6
ADP-bound	3431 ± 221	−33.7 ± 3.9	0.010 ± 0.002	4.96 ± 0.26	0.77 ± 0.01	37.9 ± 0.3	0.51 ± 0.03	45.8 ± 0.9
Two-electron reduced	4323 ± 32	−17.8 ± 0.6	0.045 ± 0.003	3.34 ± 0.08	0.70 ± 0.001	40.2 ± 0.003	0.39 ± 0.17	50.1 ± 5.9
Fully reduced	2113 ± 578	−27.1 ± 3.6	0.126 ± 0.074	2.49 ± 0.80	0.74 ± 0.03	38.7 ± 0.9	0.59 ± 0.05	43.4 ± 1.6

* The values given in the table represent the averages of the results of 2–6 individual experiments. The individual estimates of Δ*V*^0^ and *P*_½_ were obtained from fitting the pressure dependencies of the apparent efficiency of FRET (Equation (1)) with Equation (6). These estimates were used to calculate the values of *K*°_*eq*_ and Δ*G*° given in the table. The FRET efficiencies in the end states and the respective inter-probe distances were calculated from parameters *A*_0_ and A_max_ in Equation (6). The “±” values correspond to the confidence interval calculated for *p* = 0.05.

**Figure A1:**
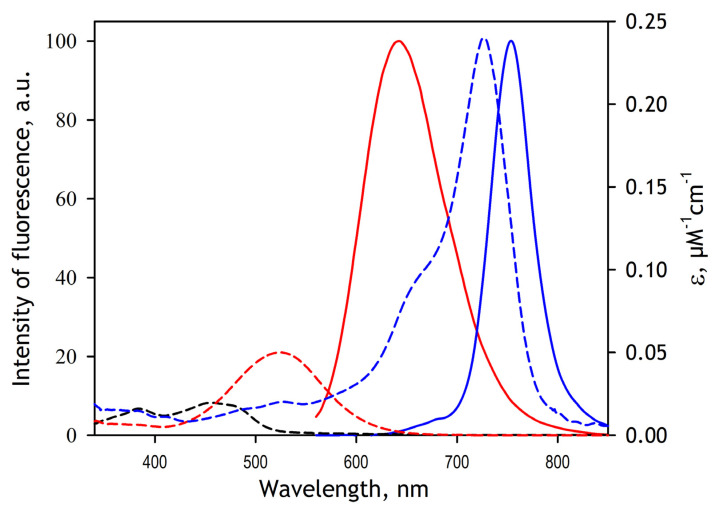
Spectra of absorbance (dashed lines) and fluorescence (solid lines) of DY520-XL (red) and DY731 (blue). The absorbance spectrum of SbCPR is shown in a black dashed line.

**Figure A2:**
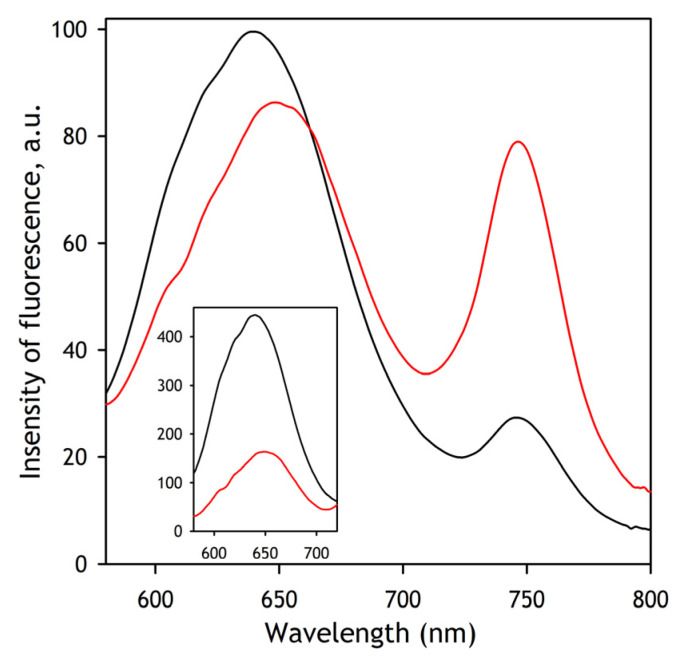
Spectra of fluorescence of SbCPR-2DY (red) and an equimolar mixture of single labeled SbCPR-DY520XL with SbCPR double-labeled with MBBR and DY-731 (black) taken at 5 °C with excitation at 505 nm. The spectra shown in the main panel are normalized to the same integral intensity of fluorescence. The inset shows the donor emission bands unscaled.

## Data Availability

Raw experimental datasets may be obtained from the authors upon a reasonable request.

## Appendix A. Design and Characterization of the Donor–Acceptor Pair for Intramolecular FRET Experiments in SbCPR

### Appendix A.1. Selection of Fluorescent Probes

To explore the conformational landscape of SbCPR and investigate the relationship between the position of the equilibrium between the open and the closed states, we used a FRET-based technique. To avoid any considerable overlap of the excitation and emission spectra of the probes with the bands of absorbance and fluorescence of CPR flavins, we employed a pair of far-red- and infrared-emitting fluorophores. As a fluorescence donor, we chose DY-520XL. This extra-large Stokes shift fluorophore exhibits a broad emission band centered around 640 nm and has its excitation maximum at 525 nm (both positions were determined in aqueous solutions). The acceptor fluorophore was represented by DY-731 dye, which has its excitation and emission maxima around 730 and 750–770 nm, depending on the environment. A vast overlap of the emission band of DY520XL with the spectrum of excitation of DY731 (Figure A1) provides for efficient FRET in this donor–acceptor pair.

### Appendix A.2. Determination of Quantum Yield of DY520-XL and the Förster Distance of Its FRET Pair with DY731

To determine the SbCPR Förster distance characteristic to our donor/acceptor pair and quantitatively interpret the results of FRET experiments, we had to estimate the quantum yields of the donor fluorophore first. To this aim, we employed the relative technique of Parker and Rees [67] using Rhodamine 101 as a quantum yield reference [68]. To determine the quantum yield of DY-520XL in its SbCPR-attached state, we measured the spectra of absorbance and fluorescence of diluted solutions of Rhodamine 101 in ethanol and SbCPR C596S variant modified with DY-520XL at 1:1 molar ratio in 20 mM Na-Hepes buffer, pH 7.4. The concentrations were adjusted to the optical density at the wavelength of excitation (520 nm) around 0.08. Two separate sets of measurements were made at 5 and 25 °C, the temperatures used in our kinetic and pressure-perturbation experiments, respectively. According to these measurements, the yield of DY520-XL attached to SbCPR at 25 °C is equal to 0.053. At 5 °C, its value increases to 0.0689.

To determine the Förster distance characteristic of the donor–acceptor pair, we recorded the fluorescence spectrum of the SbCPR C596S variant modified with DY-520XL (excitation at 505 nm) the spectrum of absorbance of the same protein modified with monobromobimane (MBBr) and DY-731. Here, MBBr, a thiol-reactive probe that does not absorb in the region of interest (520–900 nm), was used as a substitute of DY-520XL to modify the most accessible cysteine of SbCPR (Cys235) prior to attaching the DY731 dye. Using these spectra and the quantum yield of the donor determined as above, we calculated the Förster distance of the DY-520XL/DY-731 pair using PhotoChemCAD software [55]. In these calculations, we assumed the orientation factor and the refractivity coefficient to be equal to 0.6667 and 1.4, respectively. According to these calculations, the Förster distances characteristic to our pair at 5 and 25 °C are equal to 48.30 Å and 46.23 Å, respectively.

### Appendix A.3. Direct Determination of FRET Efficiency in SbCPR-2DY

To determine the FRET efficiency in the double-labeled SbCPR-2DY, we compared the intensity of fluorescence of the donor fluorophore in SbCPR-2DY with that in the equimolar mixture of SbCPR C596S variant modified with DY-520XL at 1:1 molar ratio (SbCPR-DY520XL) with the same protein modified with monobromobimane (MBBr) and DY-731 (SbCPR(MBBr, DY-731)). Here, again, we used MBBr as a substitute for DY-520XL to modify the most accessible cysteine of SbCPR (Cys235) prior to attaching the DY731 dye. The solutions were adjusted to the same concentrations of both probes. The incorporation of DY-731 into the protein results in a dramatic increase in the relative amplitude of the acceptor fluorescence band (Figure A2, main panel) at the expense of a profound drop in the intensity of the donor fluorescence (Figure A2, insert). These changes are indicative of an extensive FRET between the dies in the double-labeled protein. The ratio of the integral intensity of fluorescence of the donor in SbCPR-2DY (*I*_2DY_) to that in the mixture of SbCPR-DY520XL (*I*_mix_) with SbCPR(MBBr, DY-731) was used to determine the FRET efficiency (*E*) according to the following relationship:

$$E=1-\frac{I_{2DY}}{I_{\mathrm{mix}}}$$

The estimates of the FRET efficiency in SbCPR-2DY at 25 at 5 °C determined in this way are equal to 0.66 and 0.60, respectively.

These estimates were used to determine the quantum yield of SbCPR-incorporated DY-731, whose knowledge is necessary for estimating the FRET efficiency based on the spectra of fluorescence of SbCPR-2DY. To this aim, we used Equation (1) resolved relative to the quantum yield of the acceptor (Φ*_A_*):

$$\Phi_{A}=\Phi_{D}\frac{(1-E)\cdot I_{A}}{E\cdot I_{D}}$$

where Φ*_D_* is the quantum yield of donor fluorescence, *E* is the FRET efficiency, and *I**_A_* and *I_D_* are the integral efficiencies of fluorescence of acceptor and donor, respectively. The values of the quantum yield of DY-731 at 5 and 25 °C calculated in this way are equal to 0.0177 and 0.0158.

The knowledge of the quantum yields of the donor and acceptor allowed for estimating FRET efficiency from the spectra of fluorescence of the double-labeled protein. To this aim, we constructed the sets of the prototypical spectra of SbCPR-incorporated DY-520XL and DY-731 scaled proportionally to their quantum yields. Approximation of the spectra of fluorescence of SbCPR-2DY by a linear combination of these spectral standards was used in this study for estimating the FRET efficiency and studying its changes in rapid kinetics and the pressure-perturbation experiments.

## Appendix B. Probing the Accessibility of SbCPR Cysteines and Determining the Position of the Probes in SbCPR-2DY

In our preliminary experiments, we probed the accessibility of SbCPR cysteines for modification with the use of monobromobimane (MBBr) as a thiol-reactive probe. MBBr, which is essentially nonfluorescent until conjugated with thiol groups, readily reacts with low molecular weight thiols (glutathione, N-acetylcysteine, mercaptopurine, etc.), peptides, and proteins. The titration of MBBr-accessible thiol groups in SbCPR was performed by the sequential addition of molar equivalents of MBBr to 10 µM solution SbCPR at 4 °C. After each addition, we recorded the kinetics of the increase in fluorescence at 490 nm (excitation at 395 nm). The first two additions were followed by an increase in MBBr fluorescence, obeying the second-order kinetic equation with the rate constants of 0.008 and 0.028 µM^−1^ s^−1^, respectively. The increases in fluorescence caused by these two sequential additions were of comparable amplitudes. The addition of the third molar equivalent of MBBr at ~75% completion of the second modification resulted in a more modest and rapid increase in fluorescence, which was associated with the evident precipitation of the protein. The fourth addition did not cause any substantial fluorescence changes. The titration of the remaining unreacted MBBR with reduced glutathione revealed the presence of one unreacted equivalent of the dye. Therefore, according to these results, the protein contains three modification-accessible residues, and the modification of the third one results in protein precipitation.

The results of these preliminary experiments agree with the conclusions driven from the analysis of the SbCPR structural model. Presumably, the residues modified with the first two MBBr equivalents are Cys-235 and Cys-536, while the modification of Cys-596 becomes possible only after incorporating the first two labels and results in the protein precipitation. We validated these conclusions with MALDI-TOF mass spectroscopic analysis of the peptides generated by trypsinolysis of the MBBr-modified enzyme. When adding 3 equivalents of MBBR to the SbCPR, we can see the Cys-235, Cys-536, and Cys-596 with a mass addition of 133 compared to the unlabeled peptides.

To verify the position of the incorporated fluorophores, we subjected the unlabeled protein, its single-labeled adduct with DY520-XL, and the protein sequentially labeled with both probes to MALDI-TOF MS analysis (see Materials and Methods). Unfortunately, our experiments failed to detect the dye-modified peptides, most likely because of the large size and complex ionization modes of the modifying reagents. However, we were able to estimate the positions of the dyes from a comparison of recovery of the cysteine-containing peptides in the unlabeled protein with that in the single- and double-labeled samples. The peptides containing Cys-306, Cys-325, Cys-352, Cys-503, Cys-520, and Cys659, which are thought to be inaccessible for modification, were equally well detected in all three samples. In contrast, the peptide containing Cys-536 was recovered in all samples, but the double-labeled one, while neither of the single- or double-labeled proteins exhibited a detectable Cys-235-containing peptide. These results suggest that our sequential labeling procedure results in the protein where DY-520-XL residue is attached to Cys-235, while DY-731 fluorophore is located at Cys-536 residue.
